# Biodegradable Oligoesters of ε-Caprolactone and 5-Hydroxymethyl-2-Furancarboxylic Acid Synthesized by Immobilized Lipases

**DOI:** 10.3390/polym11091402

**Published:** 2019-08-26

**Authors:** Anamaria Todea, Ioan Bîtcan, Diana Aparaschivei, Iulia Păușescu, Valentin Badea, Francisc Péter, Vasile Daniel Gherman, Gerlinde Rusu, Lajos Nagy, Sándor Kéki

**Affiliations:** 1 University Politehnica Timisoara, Faculty of Industrial Chemistry and Environmental Engineering, Biocatalysis Group, C. Telbisz 6, 300001 Timisoara, Romania; 2 Faculty of Civil Engineering, Hydrotechnical Department, Politehnica University of Timisoara, Victoriei Sq. 2, 30006 Timisoara, Romania; 3 Department of Applied Chemistry, Faculty of Science and Technology, University of Debrecen, Egyetem tér 1, H-4032 Debrecen, Hungary

**Keywords:** copolymerization, renewable, oligoester, lipase, furan-based, ε-caprolactone

## Abstract

Following the latest developments, bio-based polyesters, obtained from renewable raw materials, mainly carbohydrates, can be competitive for the fossil-based equivalents in various industries. In particular, the furan containing monomers are valuable alternatives for the synthesis of various new biomaterials, applicable in food additive, pharmaceutical and medical field. The utilization of lipases as biocatalysts for the synthesis of such polymeric compounds can overcome the disadvantages of high temperatures and metal catalysts, used by the chemical route. In this work, the enzymatic synthesis of new copolymers of ε-caprolactone and 5-hydroxymethyl-2-furancarboxylic acid has been investigated, using commercially available immobilized lipases from *Candida antarctica* B. The reactions were carried out in solvent-less systems, at temperatures up to 80 °C. The structural analysis by MALDI TOF-MS, NMR, and FT-IR spectroscopy confirmed the formation of cyclic and linear oligoesters, with maximal polymerization degree of 24 and narrow molecular weight distribution (dispersity about 1.1). The operational stability of the biocatalyst was explored during several reuses, while thermal analysis (TG and DSC) indicated a lower thermal stability and higher melting point of the new products, compared to the poly(ε-caprolactone) homopolymer. The presence of the heterocyclic structure in the polymeric chain has promoted both the lipase-catalyzed degradation and the microbial degradation. Although, poly(ε-caprolactone) is a valuable biocompatible polymer with important therapeutic applications, some drawbacks such as low hydrophilicity, low melting point, and relatively slow biodegradability impeded its extensive utilization. In this regard the newly synthesized furan-based oligoesters could represent a “green” improvement route.

## 1. Introduction

The advancement of greener alternatives in polymer science has been afforded much attention in the past years, and this sustainability effort has continued, as both academia and industry are increasingly focused on green raw materials, green chemistry, and green processing [1]. To this end, the conversion of renewable resources into environmentally friendly and biodegradable polymeric materials, means that mainly polyesters, through enzymatic polymerization has become a particularly emerging path [2,3,4,5,6].

*Candida antarctica* Lipase B (CALB) is the most extensively studied enzyme in biocatalytic polyester synthesis [7,8,9]. Compared to many conventional catalysts, CALB can produce novel polyesters, containing sensitive structures without toxic residuals; therefore, the products have promising applications in biomedical and tissue engineering. Several bio-based monomers, such as succinate, fatty and hydroxy fatty acids from vegetable oils, γ-butyrolactone, isohexides derived from sugars, 1,4-butanediol, glycerol, have been studied [10,11,12].

Among a diversity of possible monomers, obtained from renewable resources, furan derivatives are promising raw materials for bio-based polymers, since they include an aromatic structure and can be obtained from sugars and polysaccharides [13,14,15]. It was already demonstrated that aromatic compounds provide rigidity to a polymer chain and the polymers with rigid backbones could have improved thermal and mechanical stability. Furan-based polyesters are suitable as high-performance polymers, having similar or even better properties than similar petrol-based equivalents, e.g., poly(ethylene furanoate) (PEF) shows better barrier properties than poly(ethylene terephthalate). Several furan polyesters have been synthesized, particularly from dimethyl 2,5-furandicarboxylate (DMFDCA) or 2,5-bis(hydroxymethyl)furan (BHMF), such as poly(ethylene furanoate) or poly(butylene furanoate) (PBF) [14].

5-Hydroxymethylfurfural (HMF) is the most important chemical building block, that has been utilized as a bio-based furan source. These compounds can be obtained by dehydrating fructose or glucose and then converted in several ways, depending on the reaction type of the hydroxymethyl and formyl functional groups, or of the furan ring. HMF is an important source of other furan-based platform chemicals [16,17]. Particularly, the formyl group, which may be oxidized in a selective manner, without reactions at the hydroxyl group, yielding 5-hydroxymethyl-2-furancarboxylic acid (also named 5-hydroxymethyl-2-furoic acid, HMFA) [18]. Subbiah et al. demonstrated that HMF can be very efficiently converted to 2,5-dihydroxymethylfurfural (DHMF) and HMFA via a Canizzaro reaction [19]. Although this substrate can be easily used as bio-based monomer for copolyester synthesis, combined with another cyclic or linear hydroxy acid; such processes have not yet been reported. 

ε-Caprolactone (ECL) was an important, industrially-used monomer, since polycaprolactone (PCL) belonged to the first generation of aliphatic polyesters, utilized in drug delivery systems. Although, it was later replaced in these applications by other polymeric materials, in the last years PCL has gained a renewed interest, mainly due to its slow crystallization kinetics and low melting temperatures in the physiological range, that can be exploited in the design of tunable biomaterials [20]. ECL is industrially-obtained from petroleum-based raw materials, but a biobased route from lignocellulosic material is also possible as a valuable alternative to the fossil resources [21]. An important advantage of ECL resides in the easy formation of copolymers with other monomers, which was exploited by both chemical [22] and enzymatic reaction pathways, including the utilization of furan-based co-monomers. 

Morales-Huerta et al. reported on the enzymatic synthesis of co-polyesters, prepared by ring-opening polymerization (ROP) of ε-caprolactone, with cyclic butylene 2,5-furandicarboxylate oligomers. Their approach was to design PCL co-polyesters with improved properties, using a bio-based aromatic co-monomer and applying a greener synthetic route [23].

Jiang et al. enzymatically polymerized 2,5-*bis*(hydroxymethyl)furan with various diacid ethyl esters by *Candida antarctica* Lipase B (CALB), via a three-stage method, obtaining a series of novel bio-based furan polyesters, with number-average molecular weights (*M*_n_) around 2000 g/mol. The effects of the number of the methylene units in the dicarboxylic segments, on the physical properties of the furan polyesters, were also studied [12].

The aim of this study was to synthesize and characterize oligoesters, based on ECL and HMFA, using commercially available lipases. To the best of our knowledge, it is the first report on the synthesis of bio-based oligomers derived from HMFA. One of these lipases has not been previously reported as a biocatalyst for oligoesters synthesis and could represent an alternative to the most common lipase for synthetic purposes. These oligomers may have important applications in the biomedical field, particularly for controlled drug delivery. Considering that ECL can be also obtained from renewable resources, our findings represent a completely green route to new bio-based and biodegradable oligomeric materials. The biodegradation of these oligomers was also studied for the first time, compared to PCL.

## 2. Materials and Methods

### 2.1. Materials

5-Hydroxymetyl-2-furancarboxylic acid (HMFA, >97%), was acquired from Carbosynth Compton, Berkshire, UK). ε-Caprolactone (ECL, ≥98%), tetrahydrofuran (THF, >99%), toluene (>99%), chloroform (CHCl_3_, >99%), dichloromethane (CH_2_Cl_2_, ≥99,8%), acetonitrile (>99%), sodium sulphate (Na_2_SO_4_, >99%) were from Sigma Aldrich (Steinheim am Albuch, Germany). Magnesium chloride (MgCl_2_, 98%) and potassium carbonate (K_2_CO_3_, 99%) were purchased from Chimopar S.A. (Bucharest, Romania), while sodium chloride (NaCl, >99%) and potassium sulfate (K_2_SO_4_, 99%) were products of VWR Chemicals (Leuven, Belgium) and Alfa Aesar GmbH & Co KG (Karlsruhe, Germany), respectively. All chemicals were used as purchased, without further purification.

Three commercially available immobilized enzymes were utilized as biocatalysts for the polymerization reactions: Novozyme 435 (lipase from *Candida antarctica* B immobilized on acrylic resin) and Lipozyme *Cal*B, (from *C. antarctica*) were products of Novozymes (Bagsværd, Denmark), while GF-*Cal*B-IM (lipase B from *C. antarctica* immobilized on microporous ion exchange resin) was purchased from GenoFocus (Daejeon, South Korea). Lipase from porcine pancreas (Sigma-Aldrich, Steinheim, Germany) was used for the enzymatic degradation studies. 

### 2.2. Methods 

#### 2.2.1. Enzymatic Synthesis of the Copolyesters of 5-Hydroxymethyl-2 Furancarboxylic Acid and ε-Caprolactone 

The enzymatic polymerization reactions have been carried out in a solvent-less reaction system. 0.284 g HMFA (2 mmoles) and 445 µL ECL (4 mmoles) were homogenized in 2 mL Eppendorf tubes, then 100 mg of immobilized lipase was added. The reactions were performed at different temperatures in the 40–80 °C range and a mixing frequency of 1000 min^−1^, using a Comfort Thermomixer (Eppendorf, Hamburg, Germany). At the end of the reaction tetrahydrofuran was added to the reaction mixture and the enzyme was removed by filtration. The solvent was evaporated in a vacuum drying oven at 60 °C, yielding a yellowish precipitate. The precipitate was washed 3 times with ethyl acetate to remove the PCL homopolymer and dried at 40 °C under vacuum, overnight. All reactions were carried out in duplicate and every analysis has been also made in duplicate. The presented results represent the average values, since the deviation of the individual data of the same measurement was below 5%.

#### 2.2.2. Pre-Equilibration of Water Activity

To have a controlled initial water activity, the raw materials and the immobilized lipases were equilibrated overnight with saturated salt solutions at 25 °C, in separate containers, as previously described [24]. The salts were selected to cover a broad range, from low to high water activity, as MgCl_2_ (a_w_ 0,225), K_2_CO_3_ (a_w_ 0,432), Na_2_SO_4_ (a_w_ 0,95) and K_2_SO_4_ (a_w_ 0,973) [25].

#### 2.2.3. Multiple Use of the Biocatalyst

The biocatalyst efficiency in repeated reaction cycles was investigated in the reaction conditions stated for the initial process, 24 h at 80 °C, at ECL: HMFA molar ratio of 2:1 and mixing frequency of 1000 min^−1^. The immobilized enzyme was separated by filtration at the end of each reaction cycle and washed three times with tetrahydrofuran. The solvent was removed by evaporation and the biocatalyst was used in a new batch synthesis. 

#### 2.2.4. MALDI TOF-MS Analysis 

The reaction products were analyzed by MALDI TOF-MS spectrometry, performed with a Bruker Autoflex Speed mass spectrometer (Bruker Daltonik GmbH, Bremen, Germany), which was equipped with a time-of-flight (TOF) mass analyzer. In all cases 21 kV and 9.55 kV were applied as reflector voltage 1, and reflector voltage 2, respectively. The matrix used was trans-2-[3-(4-t-butyl-phenyl) -2-methyl-2-propenylidene] malononitrile (DCTB) and potassium trifluoroacetate ionization reagent (KTFA). Calibration of the system was done with 600, 1000, and 2000 Da polyethylene glycol (PEG) solutions. 10 μL of sample (10 mg/mL) with 10 μL of DCTB solution (40 mg/mL) and 3 μL of KTFA solution (5 mg/mL) were mixed. Approximately 1 μL of this mixture was deposited onto the sample and the MS spectra were acquired in the positive ion mode. The MS spectra were processed and evaluated, using the FlexControl and FlexAnalysis software packages from Bruker (Bruker Daltonik GmbH, Bremen, Germany).

Based on the MALDI-TOF MS spectra, the average molecular weights (*M*_n_ —number average molecular weight, and *M*_w_—weight average molecular weight), as well as the dispersity *Đ*_M_ values were calculated, as indicated by Räder and Schrepp [26]. The quantitative composition of the reaction products was also estimated, based on the MALDI TOF-MS spectra [27]. Although MALDI TOF-MS is not a typical quantitative analysis method, these estimations are useful especially for comparative purposes, as it was the case in these studies. The intensity values of the K^+^ adducts of all the polymerization products, identified in each spectrum, were summed, and the total value was set as 100%. Likewise, the sum of intensity values for each type of oligoester (linear or cyclic) was calculated. Equal ionization efficiency was assumed for each oligomer present in the reaction product and their relative content was expressed as percentage, based on the total value set at 100%. 

#### 2.2.5. NMR Analysis

NMR spectra of the reaction products were recorded with a BrukerAvance III spectrometer (Bruker Daltonik GmbH, Bremen, Germany) operated at 500.0 MHz (^1^H) and 125.0 MHz (^13^C). The samples were dissolved in CDCl_3_ or THF-d_8_, and chemical shifts are given in ppm versus tetramethylsilane TMS.

#### 2.2.6. Infrared Spectroscopy (ATR FT-IR)

The FT-IR spectra were obtained in attenuated total reflectance (ATR) by using a Bruker Vertex 70 (Bruker Daltonik GmbH, Bremen, Germany), equipped with Platinium ATR, Bruker Diamond Tip A225/Q.I. 64 of co-added scans were collected in the range of 4000–400 cm^−1^ with a resolution of 4 cm^−1^. 

#### 2.2.7. Thermal Analysis

- Thermogravimetric analysis (TG)

The thermograms of the reaction products were recorded by using TG 209 F1 Libra thermogravimetric analyzer (NETZSCH-Gerätebau GmbH, Selb, Germany). The measurements were carried out in nitrogen atmosphere, in the temperature range 20–500 °C, heating rate of 10 °C/min. The data were processed with the Netzsch Proteus—Thermal Analysis program version 6.1.0. (NETZSCH-Geraetebau GmbH, Selb, Germany).

- Differential scanning calorimetry characterization (DSC)

DSC measurements were performed by DSC 204 F1 Phoenix differential scanning calorimetry (NETZSCH-Geraetebau GmbH, Selb, Germany) under nitrogen atmosphere and of 10 °C/min heating rate, in the range 20–500 °C. The initial peak temperature (*T*_i_), the final peak temperature (*T*_f_), their difference (Δ*T* = *T*_f_ − *T*_i_), the temperature at peak (*T*_pk_) and the enthalpy (Δ*H*) were collected by thermograms processing, with the Netzsch Proteus Thermal Analysis program version 6.1.0. (NETZSCH-Geraetebau GmbH, Selb, Germany).

#### 2.2.8. Molecular Modeling of the Synthesized Oligoesters

To obtain more information about the synthesized oligomers, quantum-chemical calculations were performed using the MM+ and PM3 methods, in the HyperChem program package. All structures taken in the studies were geometrically optimized with the MM+ molecular mechanics method. The structures thus obtained were subjected to a new geometric optimization with the semi-empirical PM3/RHF method, using the Pollak-Ribier criterion with a gradient RMS (root mean square) of 0.01 kcal/mol. The purpose of these theoretical investigations was to predict which oligomeric or polymeric product, having a certain structural sequence of monomeric units, would be more likely synthesized among the possible structures, based on the chemical stability values.

#### 2.2.9. Enzymatic and Microbial Degradation

- Enzymatic degradation

The samples were formulated as 1.3 cm diameter and 1 mm thickness discs, using a manual hydraulic press. The samples were placed in individual containers and incubated at 37 °C in aqueous phosphate buffer (pH = 7.4) containing 0.02% sodium azide (NaN_3_, anti-mold and antibacterial agent to avoid bacterial proliferation) and 0.02% lipase from porcine pancreas. At set-point time intervals the samples were filtered and dried at room temperature for 24 h weighed. The degree of degradation was evaluated by mass loss (W), calculated by using the following formula,

(1) $$W=\frac{W_{0}-W_{t}}{W_{0}}\times 100$$

where *W_0_* is the initial mass of the disc, and *W_t_* represents the disk mass at time *t*. Control experiments were performed for each sample by incubation in an enzyme-free aqueous phosphate buffer solution. A reference homopolymerization reaction was carried out using only ECL as a monomer, under the same reaction conditions, as described for the copolymerization reactions (paragraph 2.2.1). The number-average molecular mass of the resulted PCL homopolymer was calculated from the MALDI-TOF MS spectrum as 2226 Da. This sample was used as PCL reference for both enzymatic and microbial degradation studies.

- Microbial degradation

The samples were formulated as 1.3 cm diameter and 1 mm thickness discs, using a manual hydraulic press. The samples were placed in individual containers and incubated at 30 °C in a culture medium, with 1 mL of mixed microbial culture inoculum, obtained by sampling water from the Bega River (Timişoara, Timiş County, Romania). The culture medium used had the following composition /100 mL of water: 0.5% peptone, 0.25% yeast extract, 0.1% glucose, 1.5% agar.

At set-point time intervals the samples were filtered, dried at room temperature for 24 h, and weighed. The degree of degradation was evaluated by the degree of mass loss, calculated with Equation (1). 

#### 2.2.10. Morphological Characterization of the Polymer Samples

The optical micrographs of the homopolymer and copolymers samples were collected by using an Optic Micros Austria microscope, with 50-fold spot magnitude, before and after the enzymatic and microbial degradation.

## 3. Results and Discussion

### 3.1. Enzymatic Synthesis of the Oligoesters 

**Figure 1:**
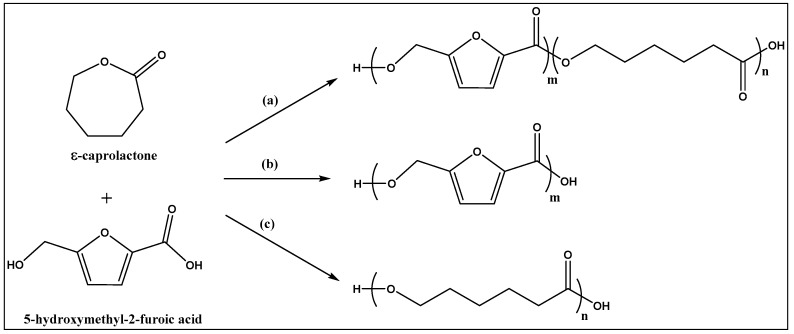
Reaction scheme for the polyester synthesis from 5-hydroxymethyl-2-furancarboxylic acid and ε-caprolactone, with formation of linear copolymer (route a) and homopolymer (route b and c) products.

In this work, oligoesters were synthesized from ε-caprolactone and 5-hydroxymethyl-2-furancarboxylic acid in a single step, and in solvent free system. The possible reaction products are cyclic and linear co-polyesters, as well as the corresponding cyclic and linear homopolymers, poly-(ε-caprolactone) and poly(5-methoxy-2-furancarboxylate) (Figure 1). In practice, the homopolymers of HMFA were not identified in quantifiable amounts among the reaction products, and this experimental detail favored the downstream process. The reactions went to completion in 24 h, since unreacted monomers were also not detected in the reaction mixture. 

**Figure 2:**
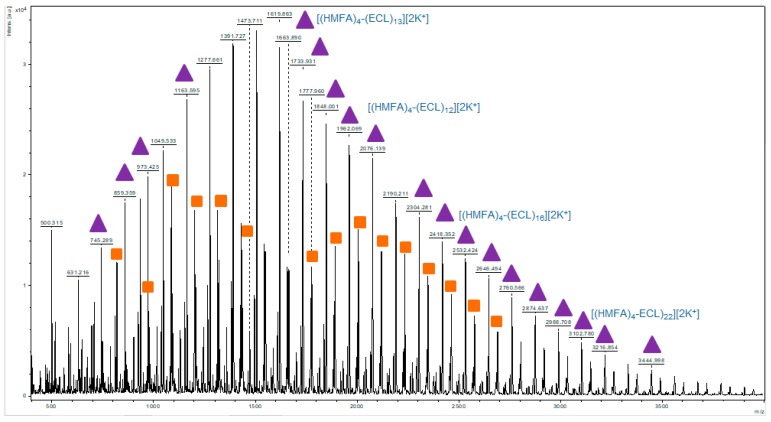
The MALDI TOF-MS spectrum of the copolymer synthesized from 5-hydroxymethyl furanoic acid (HMFA) and ε-Caprolactone (ECL) at 80 °C and 24 h reaction time, without solvent; Linear copolymers (triangles); Cyclic copolymers (squares).

The formation of the co-polyesters was demonstrated by MALDI TOF-MS spectrometry, which is considered as the most appropriate technique to prove the formation of the copolymer, since the assignment of the possible copolymer composition based on the molecular mass of different peak series from the spectrum was unambiguous. Another important advantage of this technique is the possibility of analyzing the non-purified products, since the unreacted substrates, which could be also present in the mixture in small amounts, have lower molecular masses outside the range of interest, and thus, have no influence on the results. Figure 2 shows the MALDI TOF-MS spectrum of the reaction product obtained in the presence of Novozyme 435 at 80 °C, in a solvent-less system. In the MALDI TOF-MS spectra, three main series of copolymerization products could be observed. The most intense (marked by purple triangles in Figure 2) corresponded to the linear products series (HMFA)_4_-(ECL)_1-20_. The peak values corresponded to the potassium adducts of the potassium salt (formed under MALDI conditions) of the synthesized oligomers, e.g., the peak with m/z 1962.06 corresponds to the K^+^ adduct of the copolymer containing 12 ECL units and 4 HMFA units, while the peak with m/z 3102.70 corresponded to the K^+^ adduct of the copolymer containing 22 ECL units and 4 HMFA units. Another type of identified products (marked as orange squares) corresponded to the cyclic products (HMFA)_2_-(ECL)_3-22_, e.g., the peak with m/z 1999.38 corresponded to the K^+^ adduct of the cyclic copolymers containing 2 HMFA units and 15 ECL units. The third identified series (not labeled in Figure 2), presented in smaller amounts, corresponded to the linear products (HMFA)_1_-(ECL)_3-20_. As it will also result from the experimental data (Table 1) in most cases the linear copolymer was the main product, but the presence of the cyclic copolymer was not considered as a drawback. Therefore, our synthetic strategy did not target the minimization of this product. 

Compared to the copolymers enzymatically synthesized from 2,5-furandicarboxylic acid and various diols, reported by other groups [28], where the succession of the monomeric units is inherent, the structure of copolymers obtained in this work is regulated by the specificity of the enzyme for the two monomers. The reactions were carried out using a 2:1 molar ratio ECL:HMFA, in order to use ECL as solvent for the HMFA co-monomer. Based on this molar excess, the reaction products contained a certain amount of PCL homopolymer, while the copolymers with the higher polymerization degree, contained a relatively higher number of ECL than HMFA units. The aim of this study was to investigate the formation of copolymers and to determine the relative composition of the reaction products, but it was not the goal to explore the specificity of the enzyme for the monomers in depth. 

### 3.2. Influence of the Biocatalyst and Reaction Temperature 

Three commercially available immobilized lipases from *C. antarctica* B were tested to select the most suitable biocatalyst, based on the polymerization degree and the copolyester content in the reaction product. Novozyme 435 is the most successfully used biocatalyst for the synthesis of a variety of polyesters and copolymers, while GF-*Cal*B-IM was not previously reported for the synthesis of polyesters. The reactions were carried out in the 40–80 °C temperature range, to determine the effect of the temperature on the copolymer formation and on the polymerization degree, for each biocatalyst. Because the real lipase content of these commercial products is not disclosed, the same biocatalyst/substrate ratio (100 mg/6 mmoles of total monomers) was used in all studies, based on preliminary experiments (data not shown). The relative compositions of the reaction products were calculated from the MALDI TOF-MS spectra (Table 1).

Among the tested lipases, the highest linear copolymer content at 40 °C was obtained with the GF-*Cal*B-IM biocatalyst. Lipozyme *Cal*B was the less performant, as the maximal polymerization degree of the synthesized copolymers did not exceed 10 units. However, this biocatalyst showed an important effect of temperature, since at 60 °C the maximal polymerization degree increased to 18. When Novozyme 435 was used, the increase of the temperature within the investigated range of 20 °C (from 40 to 60 °C) did not result in higher average molecular weight values, but the relative content of the copolymer exceeded 55%.

As a general tendency, the average molecular mass and the dispersity increased with the increase of the temperature, the highest values being obtained at 80 °C. However, at this temperature the relative cyclic copolymer amount exceeded 40% when Novozyme 435 and Lipozyme *Cal*B were used. The most efficient biocatalyst among the tested immobilized lipases was GF-*Cal*B-IM at 80 °C, with 60% linear copolymer content in the final product and maximal polymerization degree of 24. Even though the average molecular weight values were comparable, this biocatalyst was selected for the following studies. 

### 3.3. Influence of the Water Activity 

In polymerization reactions, lipases require very small amounts of water to maintain their catalytically active conformation [29]. Moreover, the lipases act as nucleophilic agent in the initiation step in the ring opening polymerization (ROP) reactions [30].

The water activity of the reaction mixture was controlled as described in sub-chapter 2.2.2. In this regard, the substrates and the biocatalyst were pre-equilibrated separately in a desiccator containing saturated solutions of salts whose water activity is known. Subsequently, the reactions were carried out at 40°C for 24 h. The composition of the reaction products and the dispersity values were calculated based on the MALDI TOF-MS spectra.

Pre-balancing in the absence of the saturated salt solution (probably the lowest water content) led to copolymers with the highest average molecular weights, but the composition of the copolymerization products was not significantly influenced by the water activity in the reaction system (Table 2). Although the differences were not high, the average molecular weights decreased at higher water activities, therefore, a higher water content appears to hinder the polymerization reaction.

### 3.4. Characterization of the Reaction Products by Spectroscopy and Thermal Analysis 

The reaction products were characterized by FT-IR spectroscopy, compared to the starting materials. The overlapping FT-IR spectra, corresponding to ε-caprolactone, 5-hydroxymethyl-2-furoic acid and the synthesized copolymer are shown in the Supplementary material (Figure S1). The shift of the absorption band corresponding to the carbonyl valence of 1724 cm^−1^ in ECL and 1652 cm^−1^ in HMFA to 1768 cm^−1^ in the ester was observed. 

**Figure 3:**
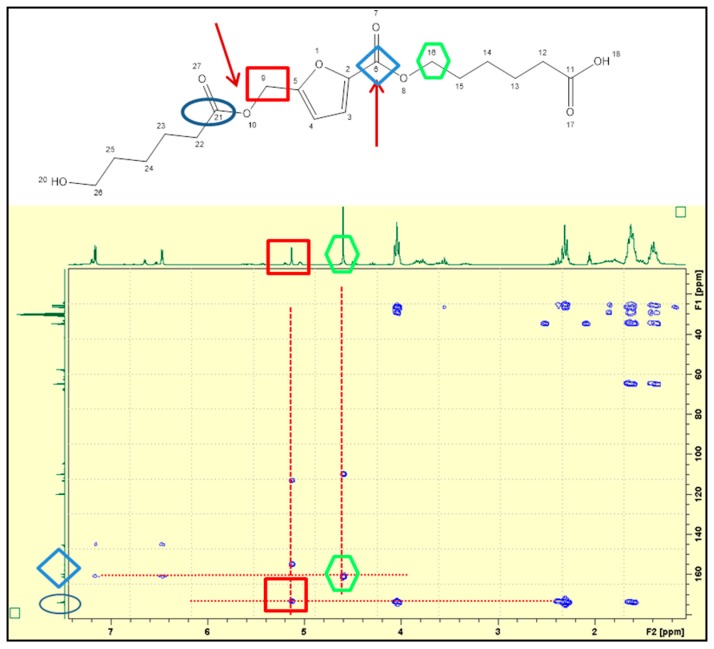
The two-dimensional 2D HMBC NMR spectra of the HMFA_ECL reaction product obtained at 80 °C, 24 h reaction time, mixing frequency 1000 min^−1^. The oligomer showed above to illustrate the spectrum is formed by two structural units derived from ε-caprolactone and one from 5-hydroxymethyl-2-furancarboxylic acid.

The formation of the co-polyester by inclusion of 5-oxymethylene-2-furanyl units in the polymeric chain was demonstrated by NMR spectroscopy. The two-dimensional 2D HMBC spectrum, shown in Figure 3, shows the remote coupling between the signals corresponding to the C21 (173 ppm) carbon atom with the signals of the C9 carbon protons (5.14 ppm), with the protons of C23 carbon (2.45 ppm) and with the protons from C24 carbon (1.6 ppm). Moreover, the coupling between he signals corresponding to the carbon atom C6 (162 ppm) with three proton signals: C16 (4.6 ppm), C3 (1.17 ppm) and C4 (6.48 ppm) can be observed. The remote coupling of C21 with protons of C9, as well as of C6 with the protons of C16 confirm the insertion of the furanic unit in the PCL backbone. The ^1^H-, ^13^C-NMR and 2D HMQC-NMR spectra (Figures S2–S4, Supplementary material) also confirm the copolymer structure.

The thermal degradation behavior and stability of polymers are of great importance for determining their potential applications [31]. The thermal stability of utilizing different co-monomers, such as adipic acid [32]. 

**Figure 4:**
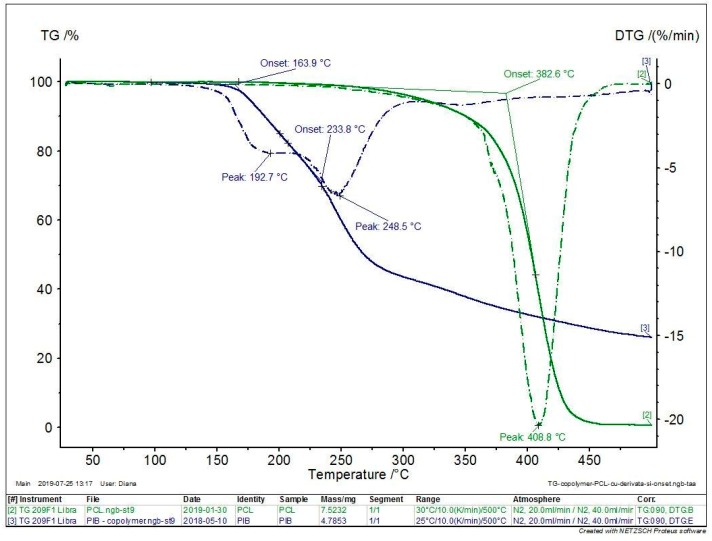
The thermograms of HMFA_ECL copolymers (blue), compared to the PCL homopolymer (green).

The thermal properties of the copolymers, compared to the PCL homopolymer and the HMFA, were evaluated by TG and DSC in a nitrogen atmosphere. The thermograms of the copolymer, PCL homopolymer, and HMFA monomer are shown in Figure 4 and the most representative decomposition parameters, such as the degradation temperatures at 5% and 10% weight loss (*T*_d,5%_, *T*_d,10%_ ), maximum decomposition temperature (*T*_d,max_ ) are presented in Table 3. The mass loss of the copolymer starts at about 170 °C. 

Compared to the PCL homopolymer for the copolymers, a decrease of *T*_d,5%_ and *T*_d,10%_ values were observed, indicating that the thermal stability of the copolymers decreased. The copolymers present a 5% weight loss above 175.8 °C. Compared to the previously reported furan-based polyesters synthesized with tetrabutyl titanate catalyst [31,33,34], the decomposition temperatures of the copolymers are comparable, about 400 °C. The copolymer degradation takes place in two steps, confirmed by two inflexion points. Comparable degradation behavior (in two steps) and degradation temperatures (*T*_d,10%_ = 240 °C, *T*_d,max_ = 390 °C) were observed by Maniar et al. for enzymatically synthesized furan-based copolymers [14].

**Figure 5:**
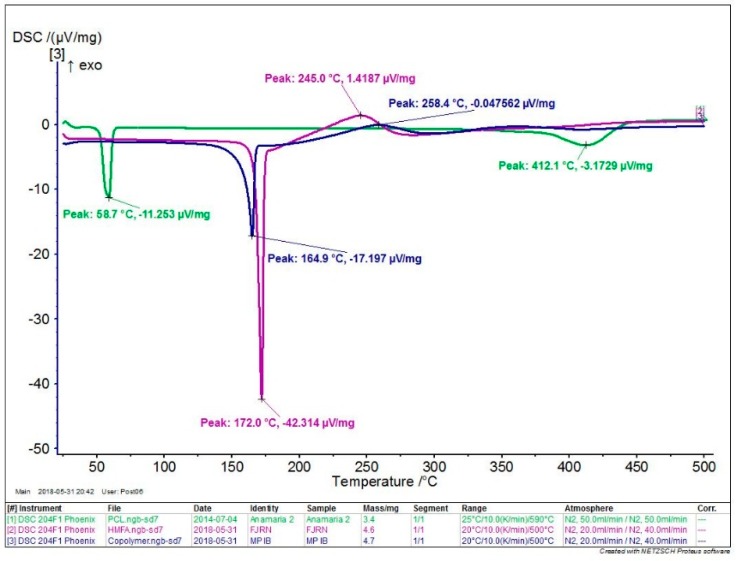
Differential scanning calorimetry characterization (DSC) curves of the HMFA_ECL (blue) copolymers, compared to PCL homopolymer (green) and HMFA monomer (purple).

The main TGA and DSC parameters of the copolymer are presented also in Table 3, compared to the PCL homopolymer and HMFA monomer. The DSC curves (Figure 5) show that the melting temperature of the copolymer is between the temperatures for HMFA and the PCL homopolymer. The copolymer exhibits an endothermic peak at 258 °C, probably due to internal transitions that may occur at degradation. The melting temperature of the copolymer is about 100 °C higher than that of the PCL homopolymer. 

### 3.5. Operational Stability of the Biocatalyst in Repeated Reaction Cycles 

The GF-*Cal*B-IM enzyme was reused in several reaction cycles at 80 °C, using the same molar ratio 2:1 of the monomers (ECL: HMFA). After a 24 h reaction, the enzyme was separated, washed three times with tetrahydrofuran, and reused in another reaction cycle in the same reaction conditions.

The average molecular weights and the relative composition of the products were calculated, based on the MALDI TOF-MS spectra. After four batch reaction cycles, the composition of the reaction product and the relative copolymer content did not change significantly, although the average molecular weights were lower (Table 4). The maximum degree of polymerization also decreased with the reutilization, indicating a gradual diminution in the catalytic efficiency of the enzyme after several cycles of use.

### 3.6. Stability Study of HMFA_ECL Copolymers by Quantum Chemical Methods 

To find out more about the monomers’ binding order, a detailed investigation of several possible structures, using the MM+ molecular mechanics method and the PM3 semi-empirical molecular orbital theory, were performed. Determining the most stable structure, at its lowest energy, provides important information about the polyester type, shape, and spatial orientation.

**Figure 6:**
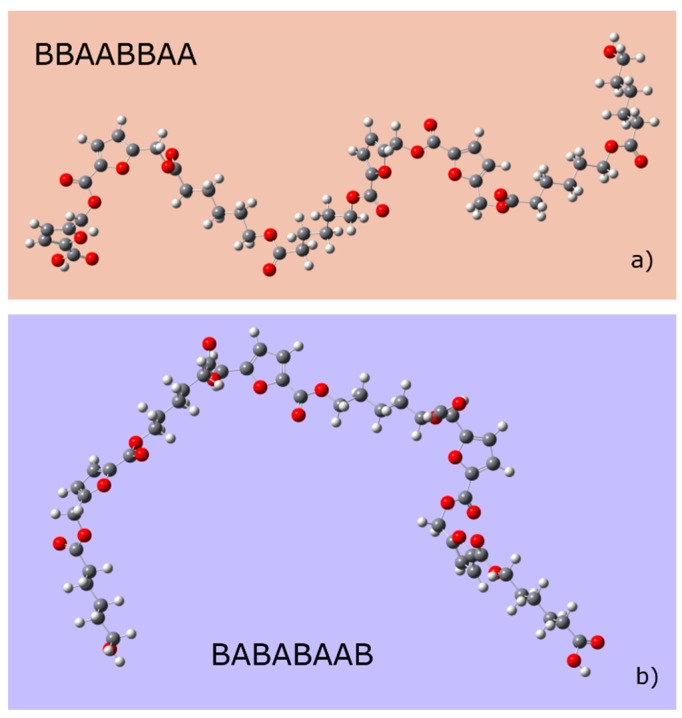
The optimized most stable structures of the linear copolymers containing four HMFA (A) and four ECL (B) monomeric units, based on the energy-stability, and (**a**) chemical reactivity (**b**). Heat formation values, HOMO, LUMO energy values, absolute hardness and chemical potential are presented in the Supplementary material, Table S2.

Based on the MALDI TOF-MS spectra, it was found that the copolymer with eight polymeric units (4 ECL units and 4 HMFA units) was present in the largest amount in most synthesized products (Table S1, Supplementary material). To determine the monomer sequence that gives the highest stability to this copolymer, all 70 possible structures, which contain four units of each monomer, were built (Table S2, Supplementary material). The method used for the geometric optimization is presented in subchapter 2.2.8. The energetically most stable structure corresponds to an ordered structure, the BBAABBAA chain, where A is ECL and B is the HMFA (Figure 6). Based on the HOMO-LUMO energy difference, the most stable structure in terms of chemical reactivity contains a rather alternately disposed sequence of ECL and HMFA units.

### 3.7. Enzymatic and Microbial Degradation of the Synthesized Oligoesters 

Determining the biodegradation of polymers is the first step before testing these materials for pharmaceutical applications. In this respect, the enzymatic and microbial degradation of the co-polyesters has been studied, compared to PCL. Two methods were selected to determine the biodegradability (i) enzymatic degradation in the presence of a lipase, and (ii) microbial degradation using a mixture of microorganisms existing in a natural environment (the Bega River, Timişoara, Romania).

The polymer samples, selected for the degradation studies, were co-polyesters of ECL with HMFA, synthesized at 80 °C with GF-*Cal*B-IM lipase as biocatalyst.

#### 3.7.1. Enzymatic Degradation with Porcine Pancreatic Lipase (PPL)

Enzymatic degradation was performed by incubating the samples at 37 °C in phosphate buffer solution (pH = 7.4) in the presence of NaN_3_ (to inhibit the proliferation of microorganisms) and porcine pancreatic lipase. Lipase catalyzes the hydrolysis of ester groups, formed by polycondensation, transforming the oligoesters into the starting materials.

**Figure 7:**
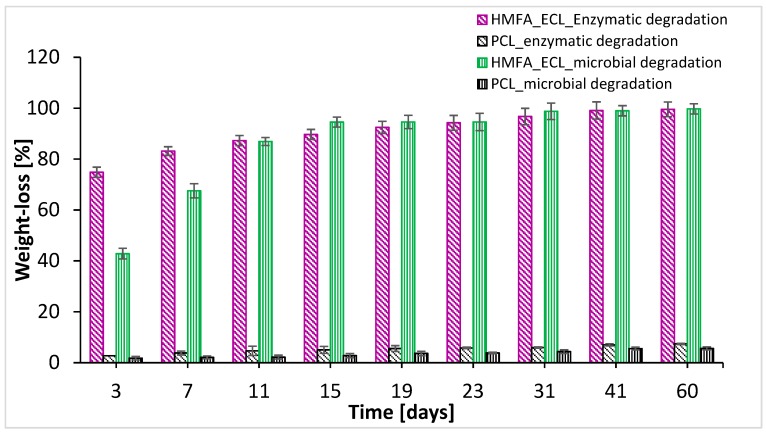
Weight losses of HMFA_ECL and PCL oligoesters after enzymatic (lipase) and microbial degradation, throughout 60 days.

The mass losses, expressed as percentage, are shown in Figure 7, which summarizes the variation in mass loss over time for the homo- and co-polymer, in the presence of both PPL lipase and microorganisms. According to Figure 7, the insertion of HMFA into the PCL polymeric chain leads to an increase in the rate of degradation of the co-polymers.

Compared to poly (lactide) or poly(glycolide) [20,35] in terms of biodegradability, a high permeability, and inability to generate an acidic environment, PCL is a better polymer concerning the potential applications in biomedicine. Moreover, due to the low PCL degradation rate, it can be used to release drugs over a longer period, even over a year [36]. PCL degradation involves the hydrolytic cleavage of ester groups. It is easy to anticipate that the insertion of different co-monomer units in the PCL chain will lead to different properties, including changes in biodegradability, thus making it possible to control the biodegradability by targeted introduction of such groups. The insertion of HMFA in the PCL chain, i.e., the presence of the heterocyclic structure in the polymeric chain, has promoted the lipase-catalyzed degradation. Three days after incubation in the presence of PPL a decrease of 74.83% was observed, related to the initial mass of the copolyester. After 19 days the mass loss was greater than 90%, and after 60 days, the copolyester with ECL and HMFA reached 99.54% degradation (Figure 7). According to the result reported by Kasmi et al., for furan based polyester degradation by *R. oryzae* and *P. cepacia* lipases, amorph polyesters are degraded faster than the the crystalline forms [34]. 

PPL was used for furan-based polyester degradation by several research groups [33,37]. The mass loss for poly(2,5-furandicarboxylate)-co-1,3-propandiol/1,4-butandiol/adipic acid, containing 30% furan units, did not exceed 30% after 28 days. Even though the degradation rate was evaluated only by the mass decrease, the present results obtained for HMFA_ECL copolymers show a better biodegradability. 

#### 3.7.2. Microbial Degradation with Microorganisms from a Natural Environment

In order to investigate the biodegradability in conditions as close as possible to the natural ones, the oligoesters were introduced into a liquid culture medium, in which microorganisms from a natural environment of flowing water (Bega river, Timişoara) were sown. These microorganisms can use polyesters as a carbon source for their growth. A natural water environment represents a unique ecosystem, with very different microbial activity and sensitivity to pollution [38].

Previous studies of other groups reported the investigation of the biodegradability of poly(ε-caprolactone) in several biotic environments, including river and lake waters, sewage sludge, farm soil, paddy soil, creek sediment, roadside sediment, pond sediment, and compost [38,39]. 

The mass loss values of the oligoesters, incubated in the presence of microorganisms, showed a difference compared to the biodegradability, which was achieved only with lipase only in the first week (Figure 7). These mass loss values are clearly superior to the PCL homopolymer, where only 5.60% of the mass was degraded after 60 days of incubation. The HMFA_ECL copolymers were better degraded, suffering a mass loss of 42.81% after 3 days of incubation in the presence of the cultivated microorganisms. After 30 days, the recorded weight loss was over 95%, and after 60 days the copolymers with furan units were almost completely decomposed (99.73%).

**Figure 8:**
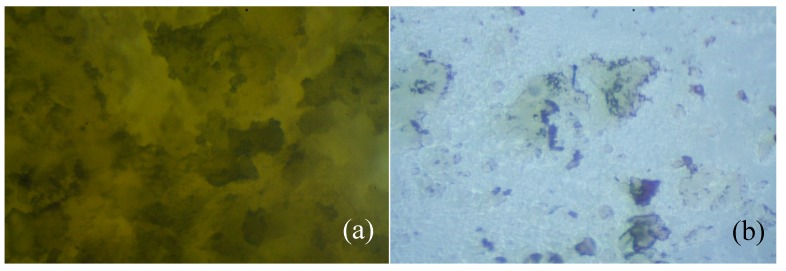
Optical micrographs of the HMFA_ECL oligomers (**a**) before and (**b**) after the microbial degradation.

The optical microscope images are also illustrative of the biodegradability of the studied oligoesters (Figure 8). The most obvious transformation is visible on the oligomer HMFA_ECL, which after 41 days of incubation with microorganisms, is almost completely degraded (approx. 1 mg remained from the initial 200 mg sample). In the case of the PCL homopolymer, only small cracks were observed, due to the action of microorganisms (not shown in Figure 8).

## 4. Conclusions

New bio-based oligomers of ε-caprolactone, with 5-hydroxymethyl-2-furancarboxylic acid, were enzymatically synthesized. Three commercially available immobilized lipases from *C. antarctica* B were tested: Novozyme 435, Lipozyme *Cal*B, and GF-*Cal*B-IM. The best results were obtained for lipase GF-*Cal*B-IM. The study of the influence of water activity on the copolymerization process demonstrated the disadvantageous effect of a higher water content on the average molecular weights of the synthesized oligoesters.

The structure of the reaction products formed was confirmed by the MALDI TOF-MS, FT-IR, and NMR spectroscopy, while the thermal behavior was evaluated by TG and DSC, indicating an improvement of the thermal properties by co-polymerization and insertion of the furan units. 

The operational stability of the biocatalyst, in four batch reaction cycles was satisfactory, with the preservation of the catalytic activity, but a gradual decrease of the average molecular weights was observed at repeated uses of the biocatalyst in the same batch conditions.

The biodegradability of the copolymers and PCL was studied in aqueous phosphate buffer at pH = 7.4 and 37 °C in the presence of porcine pancreatic lipase or of a mixture of microorganisms from a natural environment. The HMFA_ECL copolymers were degraded most rapidly than PCL. The heterocyclic structure, introduced into the PCL chain, led to higher biodegradability, which made these oligoesters good candidates for for biomedical applications.

## Figures and Tables

**Table 1:** The influence of the biocatalyst and temperature on the relative copolymer and homopolymer content of the reaction products.

Catalyst	*T* [°C]	*M*_n_ [Da]	*M*_w_ [Da]	*Đ*_M_	Relative Composition of the Reaction Product [%]				DP Max ^e^
					LC ^a^	CC ^b^	LH ^c^	CH ^d^	
Novozyme 435	40	931	1058	1.14	40.6	26.2	26.5	6.7	16
	60	1002	1085	1.08	57.5	10.6	14.5	17.3	15
	80	1280	1481	1.15	49.4	45.4	0.6	4.7	19
Lipozyme *Cal*B	40	695	745	1.07	41.7	22.9	30.2	5.2	10
	60	1113	1220	1.09	53.5	17.3	13.8	15.4	18
	80	1180	1347	1.14	52.7	40.4	0.0	6.9	19
GF-*Cal*B-IM	40	960	1085	1.13	46.9	21.1	21.9	10.2	17
	60	1128	1239	1.09	56.4	12.5	11.4	19.7	18
	80	1091	1404	1.27	60.2	24.0	11.8	3.9	24

^a^ Linear copolymer; ^b^ cyclic copolymer; ^c^ linear homopolymer; ^d^ cyclic homopolymer; ^e^ maximal. polymerization degree of the copolymer.

**Table 2:** Influence of the initial water activity on the molecular weight and composition of the copolymerization products of ε-Caprolactone (ECL) and 5-hydroxymethyl furanoic acid (HMFA). Reaction conditions: GF-*Cal*B-IM lipase, 40 °C, 24 h reaction time, mixing frequency 1000 min^−1^.

Salt	Water Activity $a_{W}^{e}$	*M*_n_ [Da]	*M*_w_ [Da]	*Đ*_M_	Relative Composition of the Reaction Product [%]				DP Max ^e^
					LC ^a^	CC ^b^	LH ^c^	CH ^d^	
-	-	891	1011	1.13	35.8	32.5	26.8	4.9	17
MgCl_2_	0.225	866	972	1.12	36.2	33.1	25.5	5.2	15
K_2_CO_3_	0.432	865	967	1.11	32.6	35.9	25.7	4.8	15
NaCl	0.750	847	941	1.11	31.2	35.9	27.7	5.2	14
Na_2_SO_4_	0.950	804	899	1.11	32.2	36.5	25.4	5.7	14
K_2_SO_4_	0.973	791	874	1.10	37.9	25.4	30.8	5.9	11

^a^ Linear copolymer; ^b^ cyclic copolymer; ^c^ linear homopolymer; ^d^ cyclic homopolymer; ^e^ maximal polymerization degree of the copolymer.

**Table 3:** Thermal properties of the HMFA_ECL copolymer, compared to the PCL homopolymer.

Sample	TGA			DSC				
	*T*_d,5%_ [°C]	*T*_d,10%_ [°C]	*T*_d,max_ [°C]	*T*_i_ [°C]	*T*_f_ [°C]	Δ*T* [°C]	*T*_pk_ [°C]	Δ*H* [J/g]
PCL	314.8	350.4	439.4	41.1	80,5	39,4	66.5	−145.5
				338.1	462.7	124.6	41.,2	−517.2
HMFA_ECL	178.5	188.3	432.8	130,4	171.4	41.0	164.9	−181.9
				214.2	294.1	79.9	258.4	123.4

*T*_d_—the decomposition temperature, *T*_i_—initial peak temperature; *T*_f_—final peak temperature; Δ*T* = *T*_f_ − *T*_i_; *T*_pk_—temperature at peak; Δ*H*—enthalpy.

**Table 4:** Reuse efficiency of the GF-*Cal*B-IM enzyme in multiple reaction cycles.

Reaction Cycle	*M*_n_ [Da]	*M*_w_ [Da]	Đ_M_	Relative Content of the Reaction Products [%]				DP Max ^e^
				CL ^a^	CC ^b^	HL ^c^	HC ^d^	
1	1091	1404	1.24	60.2	24.0	11.8	3.8	20
2	927	1034	1.12	63.3	14.9	20.7	1.0	18
3	805	896	1.13	60.9	18.6	18.0	2.5	13
4	761	823	1.08	55.4	30.6	12.2	1.8	14

^a^ Linear copolymer; ^b^ cyclic polymer; ^c^ linear homopolymer; ^d^ cyclic homopolymer; ^e^ maximal. degree of polymerization for the copolymer.

## Supplementary Materials

The following are available online at https://www.mdpi.com/2073-4360/11/9/1402/s1, Table S1: The relative amount of the copolymers at a different polymerization degree, obtained at 60 °C by using GF-*Cal*B-IM lipase as a catalyst (from MALDI TOF-MS data), Table S2: The chemical reactivity descriptors calculated for HMFA_ECL copolymers with eight monomeric units (4 ECL units and 4 HMFA units), for all possible monomeric unit sequences, Figure S1: The FT-IR spectra of HMFA monomer (black), PCL homopolymer (blue) and HMFA_ECL copolymer (red), Figure S2: 1H-NMR spectrum of the copolymerization product, Figure S3: 13C-NMR spectrum of the copolymerization product, Figure S4: HMQC 2D NMR spectrum of the copolymerization product.
