# Supplementary material for: Biodegradable Oligoesters of ε-Caprolactone and 5-Hydroxymethyl-2-Furancarboxylic Acid Synthesized by Immobilized Lipases
Source: Polymers (Basel). 2019 Aug 26;11(9):1402. doi: 10.3390/polym11091402 (PMC6780942; doi:10.3390/polym11091402)
Supplement: Supplementary file 1 [file polymers-11-01402-s001.pdf]

Supplementary material

Biodegradable oligoesters of ε-caprolactone and 5-hydroxymethyl-2-furancarboxylic acid synthesized by immobilized lipases

Anamaria Todea[1], Ioan Bîtcan[1], Diana Aparaschivei[1], Iulia Păușescu[1], Valentin Badea[1], Francisc Péter[1*], Vasile Daniel Gherman[2], Gerlinde Rusu[1], Lajos Nagy[3*], Sándor Kéki[3]

[1] University Politehnica Timisoara, Faculty of Industrial Chemistry and Environmental Engineering, Biocatalysis Group, C. Telbisz 6, 300001 Timisoara, Romania, francisc.peter@upt.ro

[2] Faculty of Civil Engineering, Hidrotechnical Department, Politehnica University of Timisoara, Victoriei Sq. 2, 30006 Timisoara, Romania

[3] Department of Applied Chemistry, Faculty of Science and Technology, University of Debrecen, H-4032 Egyetem tér 1, Debrecen, Hungary;

Table S1. The relative amount of the copolymers at different polymerization degree obtained at 60°C by using CalB IM lipase as catalyst (from MALDI-TOF MS data)

Polymerization degree	Relative amount of copolymers [%]
4	2.9
5	4.3
6	7.4
7	8.4
8	9.8
9	9.4
10	9.2
11	8.3
12	7.1
13	6.8
14	4.3
15	2.3
16	1.8
17	0.8
18	0.6

Table S2. The chemical reactivity descriptors calculated for HMFA_ECL copolymers with 8 monomeric units (4 ECL units and 4 HMFA units), for all possible monomeric unit sequences

Sample	Binding mode	Formation enthalpy [kcal/mol]	E_{HOMO} [eV]	E_{LUMO} [eV]	ΔE [eV]	η [eV]	μ eV]
1	AAAABBBB	-766.946	-9.575	-0.648	8.926	4.463	-5.112
2	AAABABBB	-765.886	-9.845	-0.736	9.109	4.554	-5.291
3	AAABBBBA	-774.103	-9.665	-0.615	9.049	4.524	-5.140
4	AAABBBAB	-773.465	-9.698	-0.557	9.140	4.570	-5.127
5	AAABBABB	-772.543	-9.628	-0.564	9.063	4.531	-5.096
6	AABABBBA	-770.886	-9.705	-0.638	9.066	4.533	-5.172
7	AABABBAB	-775.532	-9.578	-0.520	9.058	4.529	-5.049
8	AABABABB	-771.590	-9.697	-0.560	9.137	4.568	-5.129
9	AABAABBB	-772.235	-9.688	-0.597	9.090	4.545	-5.143
10	AABBBBAA	-774.648	-9.729	-0.597	9.132	4.566	-5.163
11	AABBBABA	-770.586	-9.713	-0.619	9.093	4.546	-5.166
12	AABBBAAB	-770.968	-9.683	-0.600	9.082	4.541	-5.142
13	AABBABBA	-771.334	-9.694	-0.627	9.067	4.533	-5.161
14	AABBABAB	-771.525	-9.701	-0.568	9.133	4.566	-5.135
15	AABBAABB	-769.899	-9.673	-0.584	9.088	4.544	-5.129
16	ABABAABB	-769.666	-9.678	-0.582	9.096	4.548	-5.130
17	ABABABAB	-770.296	-9.706	-0.563	9.142	4.571	-5.134
18	ABABABBA	-771.626	-9.728	-0.634	9.093	4.546	-5.181
19	ABABBAAB	-771.808	-9.676	-0.546	9.129	4.564	-5.111
20	ABABBABA	-771.311	-9.730	-0.617	9.112	4.556	-5.174
21	ABABBBAA	-770.245	-9.734	-0.587	9.146	4.573	-5.160
22	ABAABABB	-769.197	-9.624	-0.559	9.065	4.532	-5.092
23	ABAABBAB	-771.336	-9.664	-0.566	9.097	4.548	-5.115
24	ABAABBBA	-770.890	-9.694	-0.634	9.060	4.530	-5.164
25	ABAAABBB	-767.596	-9.607	-0.588	9.019	4.509	-5.097
26	ABBAABBA	-771.531	-9.666	-0.620	9.046	4.523	-5.143
27	ABBAABAB	-771.749	-9.652	-0.572	9.079	4.539	-5.112
28	ABBAAABB	-770.736	-9.627	-0.577	9.049	4.524	-5.102
29	ABBABAAB	-770.566	-9.650	-0.593	9.057	4.528	-5.122
30	ABBABABA	-770.682	-9.735	-0.603	9.131	4.565	-5.169
31	ABBABBAA	-771.301	-9.727	-0.574	9.152	4.576	-5.151
32	ABBBAAAB	-773.002	-9.675	-0.593	9.081	4.540	-5.134
33	ABBBAABA	-774.124	-9.685	-0.609	9.075	4.537	-5.147
34	ABBBABAA	-774.280	-9.728	-0.591	9.137	4.568	-5.160
35	ABBBBAAA	-774.090	-9.723	-0.594	9.129	4.564	-5.159
36	BAABAABB	-769.136	-9.662	-0.522	9.139	4.569	-5.092
37	BAABABAB	-771.725	-9.543	-0.474	9.068	4.534	-5.009

Sample	Binding mode	Formation enthalpy [kcal/mol]	E_{HOMO} [eV]	E_{LUMO} [eV]	ΔE [eV]	η [eV]	μ eV]
38	BAABABBA	-770.920	-9.639	-0.623	9.016	4.508	-5.131
39	BAABBAAB	-770.667	-9.638	-0.516	9.122	4.561	-5.077
40	BAABBABA	-770.448	-9.629	-0.623	9.005	4.502	-5.126
41	BAABBBAA	-770.080	-9.658	-0.588	9.069	4.534	-5.123
42	BAAABBAB	-769.908	-9.612	-0.531	9.080	4.540	-5.072
43	BAAABBBA	-772.411	-9.652	-0.620	9.032	4.516	-5.136
44	BAAABABB	-765.557	-9.581	-0.545	9.035	4.517	-5.063
45	BAAAABBB	-763.823	-9.559	-0.583	8.975	4.487	-5.071
46	BABBBAAA	-768.463	-9.675	-0.606	9.068	4.534	-5.140
47	BABBAABA	-771.295	-9.686	-0.600	9.086	4.543	-5.143
48	BABBAAAB	-771.377	-9.681	-0.562	9.119	4.559	-5.121
49	BABBABAA	-768.997	-9.729	-0.577	9.152	4.576	-5.153
50	BABABBAA	-771.377	-9.722	-0.591	9.131	4.565	-5.157
51	BABABABA	-770.934	-9.721	-0.625	9.096	4.548	-5.173
52	BABABAAB	-771.613	-9.724	-0.538	9.185	4.592	-5.131
53	BABAABBA	-770.795	-9.652	-0.617	9.035	4.517	-5.134
54	BABAABAB	-771.259	-9.664	-0.530	9.134	4.567	-5.097
55	BABAAABB	-770.028	-9.680	-0.646	9.033	4.516	-5.163
56	BBAAAABB	-769.234	-9.581	-0.583	8.998	4.499	-5.082
57	BBAAABAB	-765.744	-9.580	-0.540	9.040	4.520	-5.060
58	BBAAABBA	-770.139	-9.638	-0.618	9.020	4.510	-5.128
59	BBAABAAB	-766.334	-9.643	-0.562	9.081	4.540	-5.102
60	BBAABABA	-770.887	-9.660	-0.617	9.042	4.521	-5.139
61	BBAABBAA	-824.997	-9.643	-0.579	9.063	4.531	-5.111
62	BBABAAAB	-771.921	-9.675	-0.570	9.104	4.552	-5.122
63	BBABAABA	-771.918	-9.674	-0.596	9.077	4.538	-5.135
64	BBABABAA	-769.863	-9.749	-0.591	9.158	4.579	-5.170
65	BBABBAAA	-770.359	-9.737	-0.609	9.127	4.563	-5.173
66	BBBAAABA	-772.059	-9.640	-0.636	9.004	4.502	-5.138
67	BBBAAAAB	-764.611	-9.557	-0.618	8.938	4.469	-5.087
68	BBBAABAA	-772.320	-9.658	-0.592	9.065	4.532	-5.125
69	BBBABAAA	-771.568	-9.734	-0.599	9.134	4.567	-5.167
70	BBBBAAAA	-774.157	-9.602	-0.662	8.939	4.469	-5.132

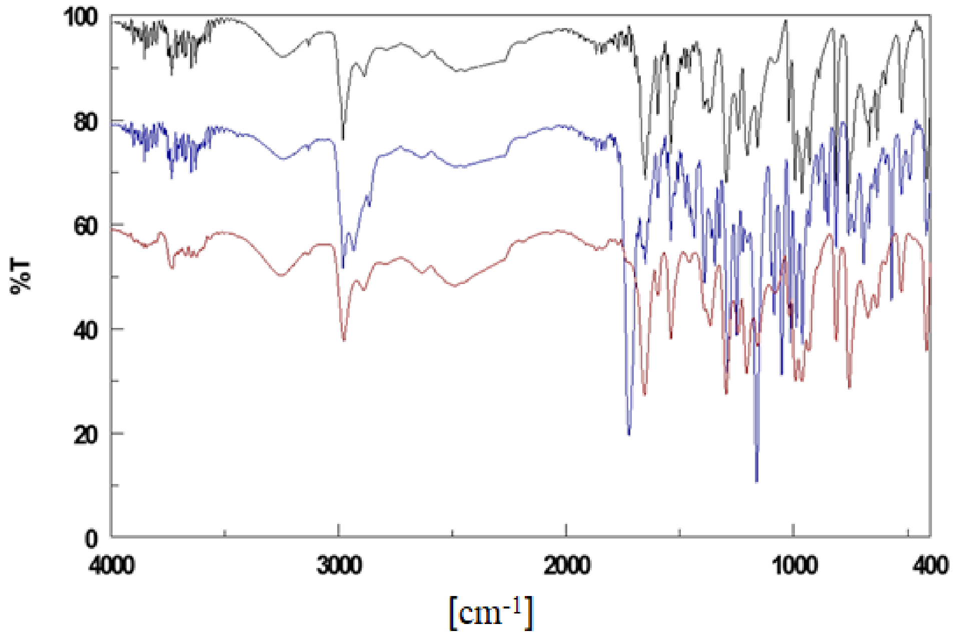

Figure S1. The FT-IR spectra of HMFA monomer (black) ECL homopolymer (blue), and HMFA_ECL copolymer (red)

In the ^{1}H-NMR spectrum of the reaction product (Figure S2), the furanic protons were assigned in accordance with the model structure presented in Figure 3 (main document) at 7.17 ppm and 6.48 ppm for the protons of the C3 and C4 atoms, respectively. The signals between 1.3 and 2.5 ppm were assigned to the (-CH_2-CH_2-) methylene protons, while the chemical shifts of the (-CH_2-O-) methylene protons were assigned between 4.05 - 4.15 ppm.

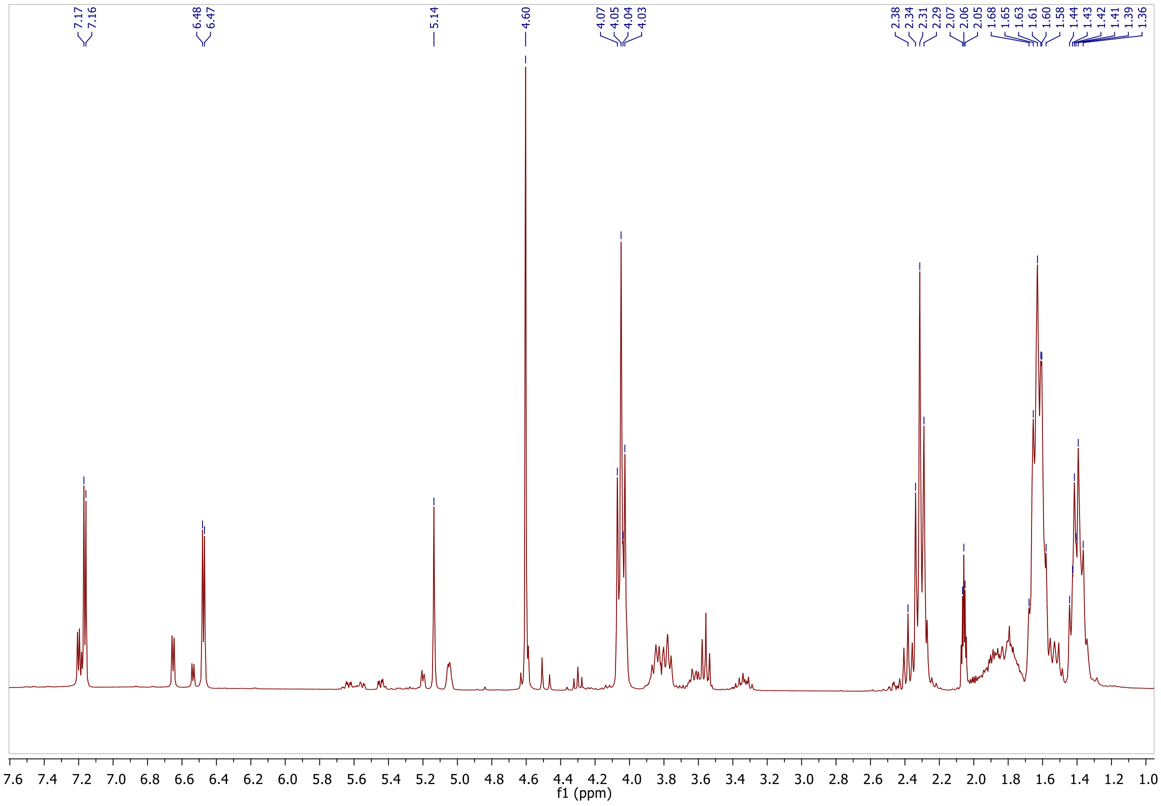

Figure S2. ^{1}H-NMR spectrum of the copolymerization product

In the ^{13}C-NMR spectrum of the reaction product (Figure S3), the methylene carbon atoms (-CH_2-CH_2-) were detected at 25 ppm (C34 from the structure presented in Figure 3), the signals at 57 and 64 ppm were attributed to the (-CH_2-O-) methylene carbon atoms, the signals of 109 ppm, 119 ppm, 162 ppm and 173 ppm were assigned to the carbon atoms C4, C3, C6 and C21, respectively (as labeled in Figure 3, main document).

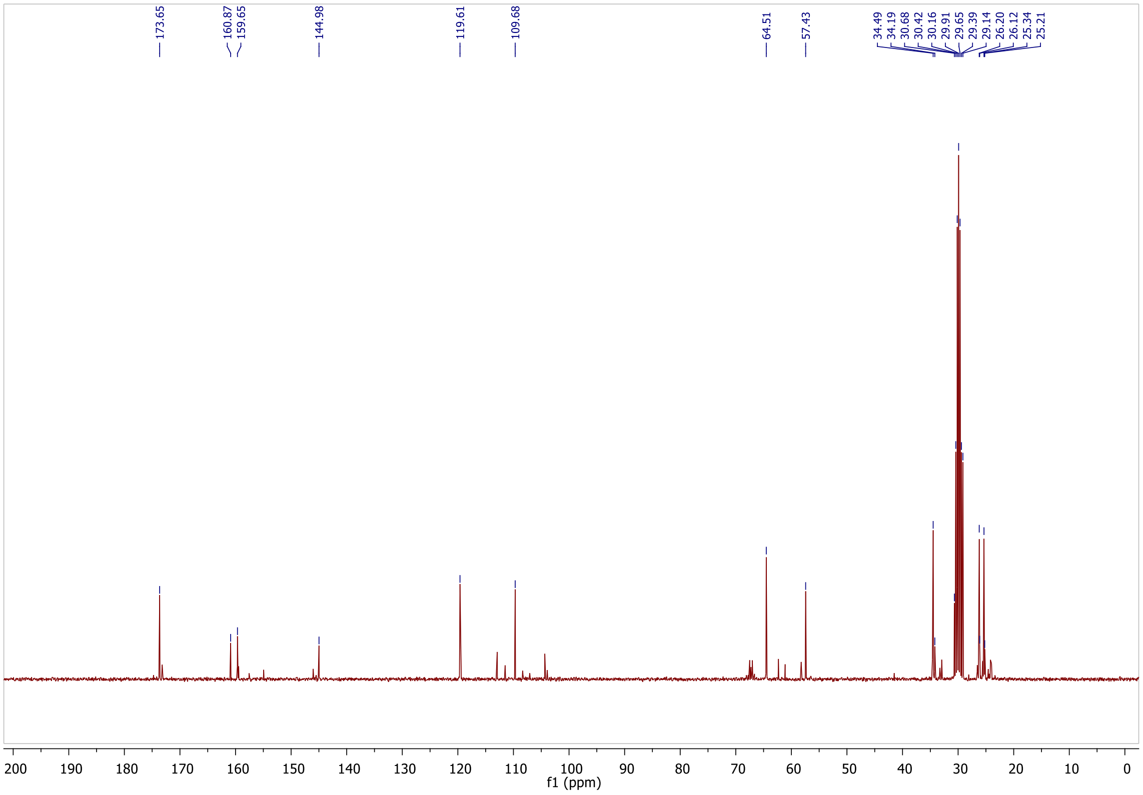

Figure S3. ^{13}C-NMR spectrum of the copolymerization product

In the 2D HMQC NMR spectrum of the copolymerization product (Figure S4) the coupling between the carbon signal from 109 ppm and proton signal from 6.48 ppm, as well as the coupling between the carbon signal from 119 ppm and proton signal from 7.17 ppm were attributed to the C3 and C4 atoms (Figure 3) of the furanic unit.

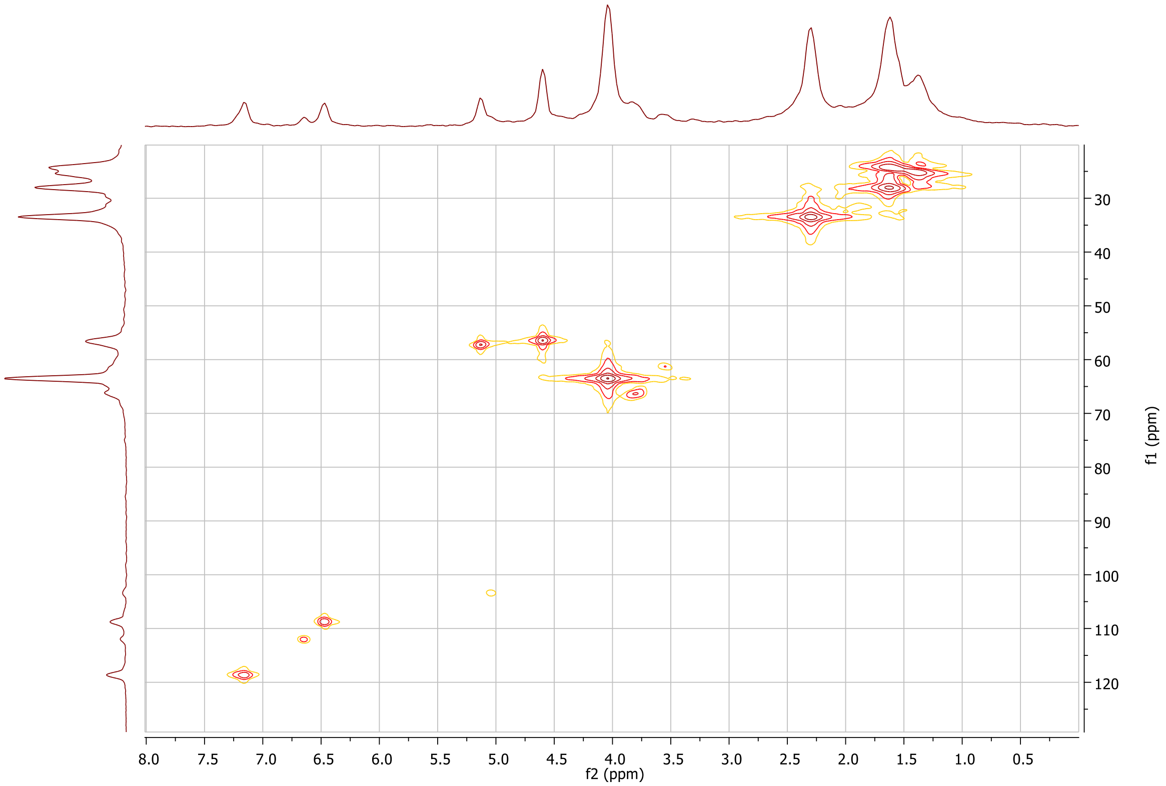

Figure S4. HMQC 2D NMR spectrum of the copolymerization product
